# Evaluation of Serological Markers in Alveolar Echinococcosis Emphasizing the Correlation of PET-CTI Tracer Uptake with RecEm18 and *Echinococcus*-Specific IgG

**DOI:** 10.3390/pathogens11020239

**Published:** 2022-02-12

**Authors:** Julian Frederic Hotz, Lynn Peters, Silke Kapp-Schwörer, Frauke Theis, Nina Eberhardt, Andreas Essig, Beate Grüner, Jürgen Benjamin Hagemann

**Affiliations:** 1 Division of Infectious Diseases and Tropical Medicine, Department of Medicine I, Medical University of Vienna, A-1090 Vienna, Austria; 2 Division of Infectious Diseases, Department of Internal Medicine III, Ulm University Hospital, D-89081 Ulm, Germany; lynn.peters@uniklinik-ulm.de (L.P.); silke.kapp-schwoerer@uniklinik-ulm.de (S.K.-S.); frauke.theis@uniklinik-ulm.de (F.T.); 3 Department of Nuclear Medicine, Ulm University Hospital, D-89081 Ulm, Germany; nina.eberhardt@uniklinik-ulm.de; 4 Institute of Medical Microbiology and Hygiene, Ulm University Hospital, D-89081 Ulm, Germany; andreas.essig@uniklinik-ulm.de

**Keywords:** alveolar echinococcosis, recEm18, PET-CTI, *Echinococcus* IgG

## Abstract

Human alveolar echinococcosis (AE), which is caused by the cestode *Echinococcus* (*E.*) *multilocularis*, is an epidemiologically relevant issue in modern medicine and still poses a diagnostic and therapeutic challenge. Since diagnosis mainly relies on imaging procedures and serological testing, we retrospectively and comparatively analyzed the performance of an *Echinococcus* IgG screening ELISA, whole serum IgE, and two specific confirmatory ELISA platforms using the defined *E. multilocularis* antigens Em2-Em18 (Em2+) and recombinant Em18 (recEm18). With special emphasis on the clinical usefulness of recEm18, we correlated the laboratory results with clinical characteristics and imaging findings in a large and well-characterized cohort of N = 124 AE patients, who were followed over several years after either surgical plus subsequent pharmacological treatment or pharmacotherapy alone. All patients had routinely received PET-CTI every two years. Our data reveal strong correlations for both *Echinococcus* IgG and recEm18 with tracer uptake in PET-CTI and parasitic lesion size and number, suggesting additional clinical usefulness of recEm18 for certain constellations only, while IgG and Em2+ still appear reasonable and sensitive screening methods for initial diagnosis of AE. With this study, we aim to contribute to further optimizing medical care of AE patients. For instance, it might be reasonable to consider the replacement of some PET-CTI follow-ups by imaging procedures with less radiation exposure or serological means alone. Further studies that clarify the correlation of serological markers with ultrasound criteria might be particularly useful, and further retrospective as well as prospective investigations are justified in this context.

## 1. Introduction

Human echinococcosis is a parasitic zoonosis caused by tapeworms of the genus *Echinococcus* (*E.*). Depending on the causative species and the clinical manifestation, it is classified as either cystic (CE, mainly caused by *E. granulosus* sensu stricto) or alveolar echinococcosis (AE, caused by *E. multilocularis*) [1,2]. Typical morphologically and pathophysiologically distinct lesions develop primarily in the liver and are eponymous for the disease as they present as either hydatid cysts in CE or poorly delimitable, infiltratively growing masses in AE [3,4,5].

Over the last decades, substantial progress has been made in the prevention, diagnosis, and management of cestode infections, including human echinococcosis [6,7]. To date, primary diagnosis of the disease is mainly based on imaging procedures and immunological testing for the confirmation of suspect imaging results, i.e., in particular, serological antibody detection in patient sera or immunohistochemistry from tissue samples. In the context of serodiagnosis, a widely applied strategy is to first screen for genus-specific antibodies to echinococcal antigens using a sensitive ELISA, followed by a further specification of the patients’ antibody responses via *E. multilocularis*-specific tests applying defined parasitic antigens that usually allow for species discrimination [8,9]. The additional benefit of total and *Echinococcus*-specific IgE quantification from sera is still being debated. Once diagnosed, every patient mandatorily receives a parasitostatic pharmacotherapy with benzimidazole derivatives (BMZ), preferably albendazole. If possible, complete surgical removal of the parasitic lesions or metacestode tissue plus temporary adjuvant pharmacotherapy is the treatment of choice [8,10], while lifelong parasitostatic pharmacotherapy with BMZ alone offers an alternative if surgery is infeasible [11,12]. Therapeutic success is again assessed using imaging and laboratory procedures. Since several serological tools are currently available for this purpose in clinical use, and as both primary diagnosis and follow-up strongly rely on serological findings, we aimed to comparatively evaluate routine test procedures for their diagnostic significance in different courses of alveolar echinococcosis. Here, we used the Serion ELISA classic *Echinococcus* IgG in primary screening for antibodies to hydatid antigens and the Bordier Em2+ as well as recEm18 ELISA for the detection of *E. multilocularis*-specific antibody responses in N = 124 clinically and radiologically well-defined AE patients who underwent different treatment regimens and follow-ups over a long-time period. The main focus of our work was on (i) their overall positive ratio in defined patient collectives and their correlation with relevant radiological and clinical parameters that are an integral part of the assessment algorithm of AE, e.g., PET-CTI tracer uptake, (ii) their performance under treatment, especially in the comparison of surgically (group A) versus pharmacologically treated individuals (group B), and (iii) their performance in follow-ups over time. We aimed to determine whether the correlation between specific serological markers and PET-CTI might justify using a modified follow-up strategy in well-defined patient subgroups. Our data further substantiate pre-existing evidence and provide new aspects concerning the clinical usefulness and possible strengths of selected serological routine markers.

## 2. Materials and Methods

### 2.1. Study Population

**Figure 1:**
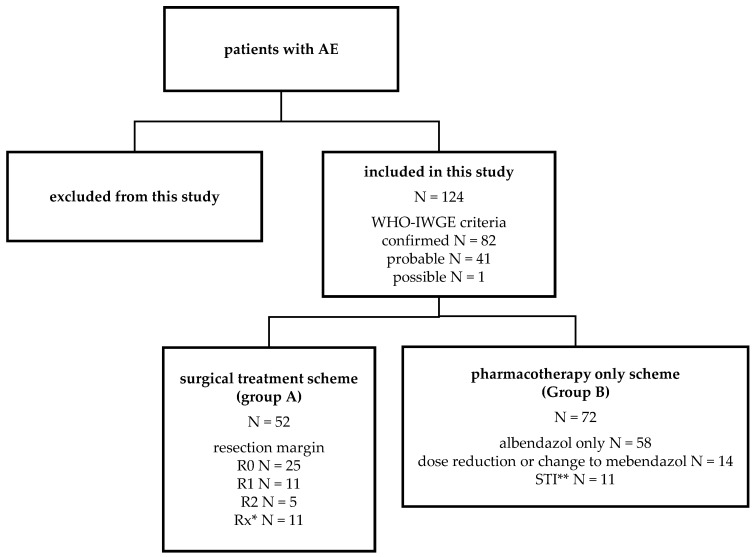
Grouping of patient collectives according to treatment schemes. Alveolar echinococcosis (AE) was classified as possible, probable, or confirmed according to the WHO–IWGE criteria [8]. * Unknown resection margin, ** structured treatment interruption.

The Ulm Working Group on Echinococcosis was established by the Department of Internal Medicine of Ulm University Hospital, Ulm, Germany in 1991. At present, roughly 700 patients with alveolar echinococcosis are under treatment or continuous observation. Of these, 124 patients with first appointment in Ulm between 1 January 2011, and 31 December 2017, as well as follow-up presentations until 31 December 2019, were included in this study. The median duration of observation was 5.7 years. The included collective (Figure 1) was divided into patients with primary surgical treatment followed by antiparasitic pharmacotherapy (N = 52, group A) and patients with antiparasitic pharmacotherapy only (N = 72, group B). Of the 52 patients in group A, 25 received a complete R0 resection (22 in domo at Ulm University Hospital), while 11 received R1 resection (9 in domo) and 5 received R2 resection (1 in domo), respectively. The resection margin of 11 individuals (2 operated in domo) was not further specified. Patients in group B received pharmacotherapy with benzimidazoles (BMZ), preferentially albendazole. In 14 patients (19.4%), there was either dose adjustment or a switch to mebendazole due to toxic drug side effects. In total, 11 patients (15.3%) had a structured treatment interruption (STI) of BMZ therapy after more than two years of continuous BMZ therapy [3]. Patients in the pharmacotherapy only group received at least two PET-CTI examinations during the time period. Patients in the surgical therapy group had at least two cross-sectional images (MRI or PET-CTI). PET-CTI was performed for all patients at Ulm University Hospital every two years. In addition, patients received abdominal sonography or MRI in the interim. Serum samples for serological investigations were taken on initial presentation and on the regular appointments for imaging follow-ups. For the study population of interest, we measured *Echinococcus* IgG, total IgE as well as the results of immune responses to specific antigens used in an Em2+ and a recEm18 ELISA.

The included population of N = 124 patients consisted of 74 female (59.7%) and 50 male (40.3%) individuals. The age of patients in group B (N = 72) averaged 66.4 ± 14.3 years and was significantly (*p* < 0.05) higher than the age of patients in group A (N = 52), who were on average 50.8 ± 17.3 years old. While patients in group A were mainly classified as stage II (32.7%), those in group B were, as expected, predominantly classified as stage IV (44.4%) [4]. Since 23 patients (16 of whom had external surgery) held no preoperative serologies, they were excluded from the calculations for the post-interventional courses of the recEm18 indices and IgG. Hence, for this purpose, the remaining patients (N = 101) were classified as group C. On their last presentation, 24 patients (46.2%) from group A were presumably cured by definition after R0 resection and a subsequent two-year period of BMZ therapy. Data of one individual (1.9%) with R0 resection were not used for outcome calculations as a two-year follow-up with BMZ had not yet been met. Therefore, the patient was considered only potentially cured. Eight patients (11.1%) from the pharmacotherapy only scheme had the best-possible outcome of being chronically stable without drugs. A total of eight patients (one patient from group A and seven patients from group B) suffered clinical progression of the disease (Table 1). 

### 2.2. Serological Testing and Imaging Procedures

Serological testing was performed at the Institute of Medical Microbiology and Hygiene at Ulm University Hospital. The institute is accredited according to DIN EN ISO 15189 and has profound clinical and technical expertise in the diagnosis of infectious diseases as evidenced by annual external quality controls. Since all tests used for the diagnostic examination of echinococcosis sera are CE-certified and since both the *Echinococcus* IgG and the Em2+ ELISA have already been validated and established in routine patient care at the time of this study, the recEm-18 ELISA had to be validated prior to use. As of now, it is used for research purposes only at our laboratory. To ensure a reproducible application, the test’s performance was validated by the determination of both intra- and inter-assay variability as well as the coefficient of variation. In short, triplicate measurements at three different instances of time from N = 10 sera of patients with histopathologically proven AE and N = 2 sera of healthy individuals were conducted. All serological findings have been critically reviewed for plausibility and reproducibility by a specialist in clinical microbiology, virology, and infectious disease epidemiology.

*Echinococcus* IgG was measured in a total of N = 305 primary and follow-up serum samples using an *Echinococcus* IgG screening ELISA (Institut Virion/Serion GmbH, Würzburg, Germany), which was carried out on a fully automated DS2 ELISA platform (Dynex Technologies, Chantilly, USA). Quantitative results were interpreted as negative (0–9 U/mL), and marginally (10–14 U/mL), weakly (15–49 U/mL), moderately (50–99 U/mL), and highly positive (≥100 U/mL), respectively. 

Em2+ ELISA (Bordier Affinity Products SA, Crissier, Switzerland) was interpreted qualitatively as positive or negative according to the manufacturer’s instructions. RecEm18 indices were calculated from the measurements of N = 456 serum samples using an *Echinococcus multilocularis* recEm-18 ELISA (Bordier Affinity Products SA, Crissier, Switzerland) according to the manufacturer’s instructions. All necessary washing steps were performed with an automated microplate washer (Biochrom Ltd., Cambridge, UK) and absorbance measurements were carried out on an Infinite F50 microplate reader (Tecan Group Ltd., Männedorf, Switzerland). 

Total IgE was measured using an electrochemiluminescence immunoassay on a Cobas e801 platform (Roche Diagnostics Deutschland GmbH, Mannheim, Germany) and results >100 kU/mL were considered positive.

The evaluation of tracer uptake in PET-CTI was performed semi-quantitatively for N = 314 PET-CTI image series assessed at Ulm University Hospital by a specialist in diagnostic radiology with experience in the imaging-based diagnosis of echinococcosis. Tracer uptake was specified as either none, weak to moderate, or strong. 

Because some patients received other imaging modalities such as MRI or sonography in addition to the PET-CTI, the evaluation of the size and the number of parasitic lesions were also based on these. Thus, a total of N = 368 imaging procedures resulted. For the size, the two largest lesions were added in two-dimensional space. On average, the lesion size at initial presentation was 69.8 ± 73.5 cm^2^ (1.1–353.4 cm^2^), and the number of parasitic lesions was 3.2 ± 2.3 (group C). 

### 2.3. Data Evaluation

Pseudonymized data were analyzed using IBM SPSS Statistics version 27. The confidence interval was set to 95% within the comparative statistics, and the significance level was set to α = 0.05. Metric variables were tested for normal distribution using the Kolmogorov–Smirnov and Shapiro–Wilk tests. For group comparisons, parametric tests were used if data were normally distributed, while nonparametric tests were used if normal distribution could not be assumed. While Student’s *t*-test with dependent or independent samples was used for parametric testing, the Wilcoxon and sign tests for dependent variables and the Mann–Whitney U test for independent variables were used as nonparametric statistical tests. 

## 3. Results

### 3.1. recEm18 Improves the IgG-Based Serodiagnosis of Metastasis-like AE Patterns

Since immunohistochemically confirmed and clinically well-characterized AE cases provide the soundest basis for the evaluation of a serological test performance, the positive ratio for *Echinococcus* IgG, Em2+, recombinant Em18, and total IgE on initial presentation was determined for N = 59 reference patients with histopathologically confirmed AE (of group C). Of these, eight (13.6%) exhibited metastasis-like imaging patterns according to EMUC-US and CTI classification [13]. Table 2 shows that *Echinococcus* IgG had the highest positive ratio of 93.2% among all serological parameters of interest addressed in this study. Interestingly, three of the four patients with negative test results for *Echinococcus* IgG were classified as having a metastasis-like pattern. By contrast, 5 of the 55 patients testing positive (9.1%) were also classified as metastasis-like. Thus, 62.5% of patients with confirmed AE and a metastasis-like appearance were successfully detected through *Echinococcus* IgG.

Em2+ and recEm18 ELISAs revealed identical positive ratios of 79.7% for the group of N = 59 patients with confirmed AE and thus recEm18 does not provide a substantial advantage over Em2+ at first glance. However, of those who tested negative for anti-Em2+ and anti-recEm18 antibodies, seven (58.3%) and five (41.7%) patients were classified as metastasis-like AE. By contrast, of those patients with positive test results, one patient (2.1%) with a positive Em2+ ELISA and three patients (6.4%) with a positive recEm18 ELISA had metastasis-like hepatic AE lesions. Thus, Em2+ ELISA detected 12.5% of individuals with a metastasis-like pattern, while recEm18 ELISA detected 37.5%, respectively.

### 3.2. Echinococcus IgG and recEm18 Correlate with Tracer Uptake, Size, and Parasitic Lesion Number

For guided decision making, it is essential to integrate clinical, imaging, and serological findings. We therefore compared established clinical, imaging-based, and serological markers, focusing primarily on *Echinococcus* IgG and recEm18 indices. For this purpose, initial assessments and follow-up imaging techniques (PET-CTI, MRI, or sonography) were considered. Both IgG and recEm18 indices showed significant (*p* < 0.05) correlations with tracer uptake in PET-CTI. These correlations were higher for the recEm18 index (K_recEm18_ = 0.660) and total *Echinococcus* IgG (K_IgG_ = 0.670) compared to the established Em2+ ELISA (K_Em2+_ = 0.556). Since both inadequately low and hyper-IgE responses are described in AE patients, total IgE expectedly showed the lowest correlation (K_IgE_ = 0.394). Furthermore, significant (*p* < 0.05) correlations were found with the size and number of parasitic lesions on imaging for IgG, Em2+, and recEm18 indices (Table 3). The recEm18 index showed the highest correlation with parasitic lesion size (K_recEm18_ = 0.711), while IgG correlated best with the number of parasitic lesions (K_IgG_ = 0.287). According to the clinical assessment, the recEm18 index had the highest correlation with a patient’s outcome (K_recEm18_ = 0.527), while IgG correlated well with the staging of the patients on initial admission (K_IgG_ = 0.321). 

Of the 59 patients with confirmed echinococcosis, 58 underwent their first PET-CTI at first presentation. Qualitatively, 86.2% of those showed a tracer uptake (N = 50). Three patients without tracer uptake had a positive recEm18 index, and six patients with tracer uptake were negative. Additionally, six had no tracer uptake in PET-CTI, but positive IgG serology, three of which were classified as a metastasis-like pattern. Furthermore, two patients with tracer uptake did not have an IgG correlate. In total, PET-CTI detected 50% of lesions with a metastasis-like pattern. The combination of total *Echinococcus* IgG and recEm18 index revealed a positive rate of 94.9% (N = 56/58). Here, 75% of all lesions with a metastasis-like pattern were covered. Only one (2.0%) of the 50 patients with tracer uptake in PET-CTI showed negative findings even if IgG and recEm18 index were together examined. By contrast, the combination of IgG and Em2+ covered only 62.5% of metastasis-like patterns and showed the same positive rate.

**Figure 2:**
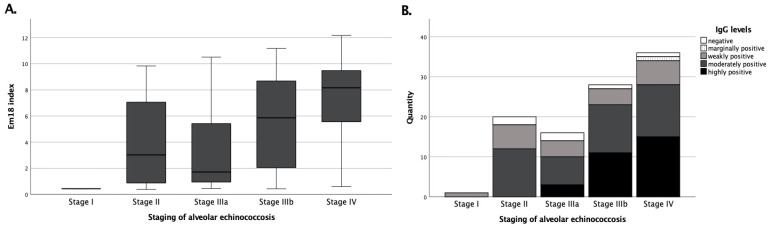
Correlations of recEm18 indices and total *Echinococcus* IgG depending on the initial staging. Patients of group C (N = 101) were included. (Panel **A**) shows the stage-dependent recEm18 indices. Accordingly, (panel **B**) exhibits the number of semi-quantitative detections of *Echinococcus* IgG depending on the patients’ staging.

When considering the individual parameters based on the staging of the 101 patients from group C, it was found that stage IV patients had the highest values for both IgG and recEm18 indices (Figure 2). The mean recEm18 index in stage IV was 7.30 ± 3.10 (median, 8.16), which was significantly (*p* < 0.05) higher than the recEm18 indices from stages IIIb (mean, 5.44 ± 3.53), IIIa (mean, 3.29 ± 3.20), and II (mean, 3.89 ± 3.44). The only patient in stage I had a recEm18 index of 0.44. 

### 3.3. Surgical Treatment Success Is Best Reflected by IgG and RecEm18 Follow-Ups

**Figure 3:**
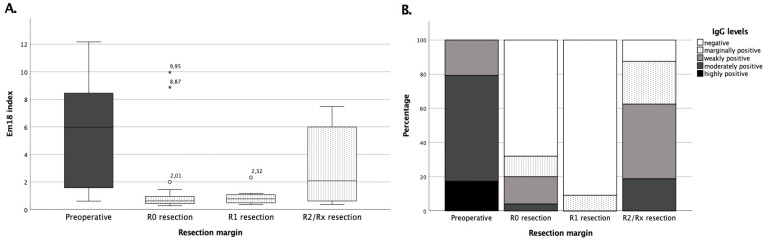
Levels of pre- and postoperative recEm18 indices depend on the resection margin. (Panel **A**) shows the recEm18 indices of group A both preoperatively and after resection depending on the resection margin, while (panel **B**) exhibits the percentage of semi-quantitative total *Echinococcus* IgG levels pre- and postoperatively according to the resection margin after surgical treatment. Serological controls were performed on average 1.9 ± 0.6 years after R0, 2.2 ± 0.2 years after R1, and 1.6 ± 2.3 years after R2/Rx resection.

Preoperatively, group A patients showed a mean recEm18 index of 5.4 ± 3.7 (median 6.0, N = 29, Figure 3) with a positive rate of 79.3%. After R0 resection, recEm18 indices dropped to a median index of 0.63 (N = 25). Two remarkably high recEm18 indices of 9.95 and 8.87 were measured in patients who had external R0 resections before the first serologies were taken at Ulm University Hospital, so their initial pre-interventional recEm18 index value remains unknown. On average, the recEm18 indices became negative after R0 resection within 2.1 ± 0.7 years (0.9–4.1), with a median of 2.0 years. After R1 resection, recEm18 indices decreased to an average of 0.88 ± 0.56 (median 0.77, N = 11) and were in general significantly (*p* < 0.05) higher after R2/Rx resection with an average of 2.98 ± 2.68 (median 2.08, N = 16) compared to R0 or R1 resection. 

Genus-specific serum IgG levels were semi-quantitatively assessed and preoperatively considered highly positive in 17.2% (N = 5), moderately positive in 62.1% (N = 18), and weakly positive in 20.7% (N = 6) (Figure 3). Preoperatively, no patient from group A had negative serologic findings for *Echinococcus* IgG (range 17.7–258.8 U/L). After R0 resection, 4.0% were still moderately positive (N = 1), 16.0% weakly positive (N = 4), and 12.0% marginally positive (N = 3). In total, 68.0% (N = 17) of the serum samples were negative. Again, three patients that were surgically treated ex domo showed higher levels with one moderately and two weakly positive IgG measurements. After R1 resection, there was a significant (*p* < 0.05) decrease in IgG levels and only one patient still showed a marginally positive result. After R2/Rx resection, 12.5% (N = 2) of serum samples showed negative IgG results, 25.0% (N = 4) were marginally positive, 43.8% (N = 7) weakly positive, and 18.7% (N = 3) moderately positive. Seven of these 16 serologies still had positive IgG values in the last presentation. 

### 3.4. Pharmacological Treatment Success Is Not Sufficiently Reflected by Serological Means Alone

**Figure 4:**
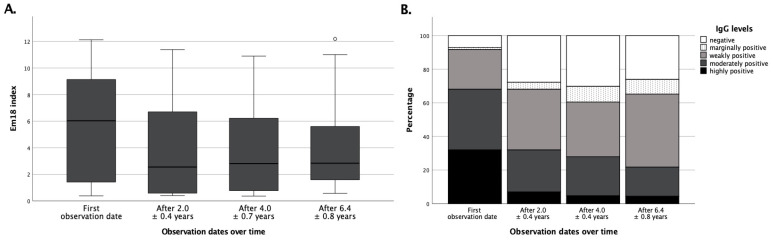
RecEm18 index and *Echinococcus* IgG under benzimidazole therapy over time. (Panel **A**) shows boxplots for patients of group A at different instances of time during follow-up. The mean recEm18 indices at the respective time points were 5.38 ± 3.70 (median 6.03, N = 72) on the first appointment; 3.72 ± 3.40 (median 2.55, N = 72) after 2.0 ± 0.4 years; 3.72 ± 3.31 (median 2.80, N = 43) after 4.0 ± 0.7 years; and 4.16 ± 3.52 (median 2.83, N = 23) after 6.4 ± 0.8 years. (Panel **B**) shows the portions of semi-quantitative IgG levels over time for patients of group A. In total, 31.9% were highly positive, 6.94% negative (N = 72) on the first appointment; 6.9% highly positive, 27.8% negative (N = 72) after 2.0 ± 0.4 years; 4.65% highly positive, 30.2% negative (N = 43) after 4.0 ± 0.7 years; 4.4% highly positive, 26.1% negative (N = 23) after 6.4 ± 0.8 years, respectively.

The 72 patients of the pharmacotherapy only scheme had an average recEm18 index of 5.38 ± 3.70 (0.40–12.13, Figure 4), with a median of 6.03, at the time the first cross-sectional image was performed. After initiation of drug therapy, there was a significant (*p* < 0.05) decrease in recEm18 indices to an average of 3.72 ± 3.40 (0.40–11.40, median 2.55) after 2.0 ± 0.4 years. However, no further significant (*p* = 0.10, *p* = 0.21) decrease of recEm18 indices was observed over time. In total, 31.9% of the IgG values were highly positive on the first appointment, while this portion significantly (*p* < 0.05) decreased to 6.94% after initiation of albendazole therapy. Subsequently, there were no further significant changes in IgG levels over time.

### 3.5. Use of Echinococcus IgG and Em2+ Is Reasonable for Initial AE Serodiagnosis

At the end of the observation period, patients clinically classified as progression (N = 8) showed significantly (*p* < 0.05) higher recEm18 indices than patients of other clinical classifications with 3.75 ± 2.69. 

**Figure 5:**
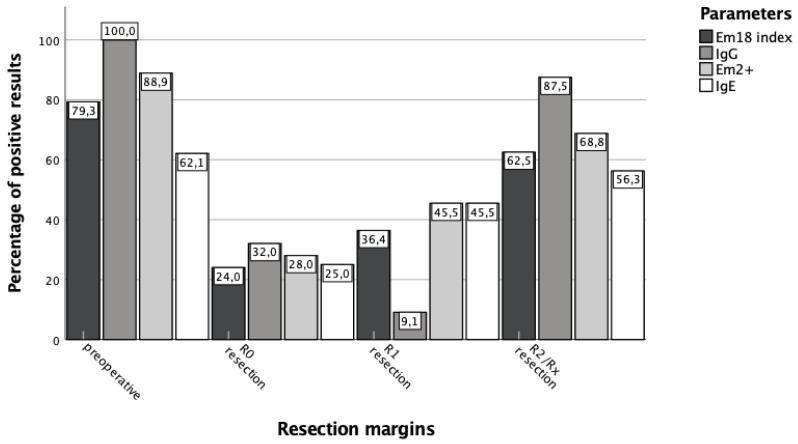
Percentage of positive results of all parameters depending on different resection margins. The graph shows the percentage of positive test results of group A pre- and postoperatively according to the resection margin (R0, R1, and R2/Rx resection). Preoperatively, recEm18 index, IgG, and total IgE were measured in N = 29, while Em2+ ELISA results were collected for N = 27 patients. Postoperatively, all parameters were determined in parallel from all serum samples, so no differences in the respective number of test results came about.

Considering the positive rates of all the parameters of group A, the pre-interventional positive rate was 79.3% for the recEm18 index, 100.0% for *Echinococcus* IgG, 88.9% for Em2+, and 62.1% for total IgE (Figure 5). Pre-interventionally, the portion of positive test results was significantly (*p* < 0.05) higher for *Echinococcus* IgG than for the recEm18 index. There was no significant difference between recEm18 index and Em2+ (*p* = 0.07). The positive rates for the recEm18 index and the Em2+ ELISA were significantly (*p* < 0.05) higher than for total IgE. *Echinococcus* IgG also showed the highest percentage of positive results after an R0 and R2/Rx resection.

## 4. Discussion

In this retrospective study, we investigated the serological markers *Echinococcus* IgG, recEm18, Em2+, and total IgE in a study population of a total of N = 124 clinically well-characterized patients diagnosed with and treated for alveolar echinococcosis (AE). We focused mainly on the positive rates of *Echinococcus* IgG, total IgE, Em2+, and recEm18 in patients with immunohistochemically confirmed AE and correlated the imaging, staging, and clinical outcomes with the respective serological test results. Furthermore, we investigated the influence of surgical and pharmacological treatment depending on surgical outcomes as defined by the resection margin and time after onset of treatment with special emphasis on recEm18 results. We provide data on the correlations and thus the clinical usefulness of the serological routine parameters mentioned with clinical aspects of the disease, and aim to further optimize the diagnostic algorithms concerning both initial diagnosis and individual follow-ups in defined AE patients.

## 4.1. Echinococcus IgG and recEm18 Are Robust Tools for Serodiagnosis of AE

A common practice for the serodiagnosis of AE is the primary screening for genus-specific antibodies using a sensitive ELISA, followed by further specification via *E. multilocularis*-specific tests applying defined parasitic antigens. Manufacturers give sensitivities/specificities for the Virion/Serion *Echinococcus* IgG ELISA of >99%/97.1% and for the Bordier Em2+ ELISA of 83%/98%, respectively. Due to the small number of patients initially evaluated for the recEm18 ELISA, Bordier provides neither data on sensitivity nor specificity in the manufacturer’s instructions. However, the performance of both commercially available and in-house recEm18 platforms has been investigated by several groups and shows sensitivities ranging from 80 to 92% [14,15]. Unsurprisingly, we found similar frequencies of positive results in a group of N = 59 patients with confirmed AE, with 93.2% for the Virion/Serion *Echinococcus* IgG ELISA and likewise 79.7% for both the Bordier Em2+ and recEm18 ELISA. Remarkably, of N = 8 / 59 patients with a metastasis-like AE pattern [13], *Echinococcus* IgG only identified 62.5% of these patients, while both specific tests performed even worse, although recEm18 (37.5%) seems to provide an advantage over Em2+ (12.5%). Since the latter is a laminar membrane antigen, it is reasonable to speculate that the total amount of laminar membrane throughout the entirety of lesions is simply not sufficient to trigger an immune response that could adequately and reproducibly be discovered by routine serological means. In contrast, Em18 was described as a viability-associated antigen [16,17,18]. Consequently, probably due to an ongoing immunological stimulus by viable echinococcal masses, we saw higher recEm18 indices and *Echinococcus* IgG with an increase in the number and size of AE lesions, and thus disease progression at primary staging. Although AE is often scattered throughout the liver in multiple smaller lesions, one would still expect positive anti-recEm18 results for a significant proportion of patients with metastasis-like AE patterns. However, the sensitivity of the commercial recEm18 ELISA might not yet be sufficient under some circumstances. In this context, immunoblotting has been examined by different groups as an alternative or additional method for the serodiagnosis and monitoring of AE and showed surprisingly high sensitivities [15,19], rendering it an ideal marker to diagnose AE. It might therefore be reasonable to apply additional immunoblotting for patients with suspected metastasis-like AE of the liver and initially negative serological findings in ELISA platforms, especially since atypical imaging results hinder proper interpretation of the findings, both in the initial diagnosis and the evaluation of regression or progression under treatment [20]. 

### 4.2. Correlation of RecEm18 and PET-CTI Tracer Uptake May Offer Alternative Follow-Up Strategies

In this study, we found a strong correlation between immune responses to recEm18 and PET-CTI tracer uptake that corroborates existing data from the literature (see above). Negativization of the Em18 serology has been described to indicate a favorable or curative AE course under treatment [15], which especially makes sense for patients who have undergone radical surgery in which all parasitic tissue has been removed. As a matter of fact, it is harder to predict the clinical course of patients under pharmacotherapy alone without radical surgery. In this context, Husmann et al. showed that PET-CTI may help determine treatment duration both in patients with resected AE and those with inoperable AE and suggest negative PET-CTI results in combination with no detectable levels of anti-Em18 antibodies to allow for safe discontinuation of benzimidazole therapy [21]. Given their correlation, it is not unexpected to see negativization of recEm18 serology in parallel with negative PET-CTI findings. However, it is worth noting here that a higher PET-CTI tracer uptake is mainly thought to be due to the activity of immune cells rather than due to parasite metabolism [22], which in itself is consistent with the fact that negative PET-CTI does not always necessarily result in negative serology [23], regardless of possible immunological reasons [24]. Especially in combination with *Echinococcus* IgG, 98.0% of all confirmed AE patients with tracer uptake in PET-CTI were detected serologically. Furthermore, six of the eight patients without any tracer uptake were identified by these serological markers.

Still, our data on the correlation of PET-CTI and recEm18 results complement recent literature, so that we believe that it appears justified to ponder if, especially for follow-up investigations on treatment evaluation in young patients, serological means plus alternative imaging procedures with less or no radiation exposure (e.g. MRI) could replace PET-CTI if combined with recEm18 and IgG serology. Here, the critical information on parasite viability could possibly be extracted from recEm18 serology while information concerning the host organs affected could be gained by imaging. Since serological markers have inherent shortcomings, e.g., in sensitivity or specificity, and depend both on initial test results at primary diagnosis and a patient’s immune status, additional imaging procedures will certainly remain indispensable for initial diagnosis as well as follow-ups in any case. For instance, an Em18 follow-up required initial seropositivity, which according to our data, holds true only for 79.7% of confirmed AE cases, a rate similarly described by other groups [15]. Analogically, an Em2+ ELISA may remain positive, if inactive calcified lesions are not entirely removed since the laminar echinococcal antigen would still be present [25,26]. 

### 4.3. Serological Follow-Ups Are Useful after Surgical Treatment, but Limited for Pharmacotherapy 

Another relevant issue is that, even for initially seropositive patients, serological follow-ups only generate limited information on disease control depending on the therapeutic strategy applied. Compared to Sulima et al. [19], we found similar ranges of negativization for the Em2+ ELISA in patients who have undergone radical surgery (72% vs. 81%) or palliative/nonradical surgery (31% vs. 20%) and could also confirm that negativization was expectedly absent in patients under pharmacological treatment only. Depending on the resection margin after surgery, we could also show a clear correlation with Em2+ seropositivity. While preoperatively 88.9% had a positive Em2+ ELISA result, rates declined to 68.8% for R2/Rx, 45.5% for R1, and 28.0% for R0 resection. It is important to stress that the recEm18 ELISA performed similarly and that the results did not significantly differ (preoperative 79.3%, R2/Rx 62.5%, R1 36.4%, R0 24.0%, respectively). According to our understanding, it therefore remains uncertain for our patient clientele whether recEm18 has a substantial advantage over Em2+ for follow-up examinations under routine conditions or whether its actual strength lies in the power to increase serological specificity and thus to discriminate more accurately between *E. granulosus* and *E. multilocularis* infections. Given that in any other than an R0 situation, parasitic antigens still remain inside a patient, it makes sense that serological negativization could not be expected through surgical intervention or pharmacotherapy alone. 

## 5. Conclusions

In summary, we have shown that there is a strong correlation between serological markers and imaging findings. Of particular interest, the combination of specific *Echinococcus* IgG and recEm18 performed better in the primary diagnosis of active AE than PET-CTI alone and was also useful in diagnosis of metastasis-like AE patterns that might reflect early viable AE lesions. Therefore, we suggest combined testing for specific *Echinococcus* IgG, Em2+ and anti-recEm18 as an initial serological screening in any patient if AE is suspected. While *Echinococcus* IgG and Em2+ seem to be sensitive markers for early diagnosis, recEm18 may provide advantages for the diagnosis of patients with metastasis-like AE patterns. Since Em18 is considered a viability-associated antigen, it might also have the potential to improve the follow-up management of a defined patient subpopulation by reducing the application of PET-CTI. In this context, the correlation of *Echinococcus* IgG and recEm18 with PET-CTI tracer uptake may justify the modification of follow-up strategies in terms of the frequent use of PET-CTI in inoperable AE patients. It may be reasonable to omit or replace PET-CTI in those patients who are only treated with BMZ by other cross-sectional imaging methods to reduce cumulative radiation exposure and follow-up costs. 

## Figures and Tables

**Table 1:** Demographic data.

	Group A Surgical Treatment Scheme	Group B Pharmacotherapy only Scheme	Group C Patients with Preoperative Serology
**N** (% of total)	52 (41.9%)	72 (58.1%)	101 (81.5%)
**age at the end of follow-up** range	50.8 ± 17.3 years 23.0–82.8	66.4 ± 14.3 years 22.8–89.0	60.8 ± 17.4 years 22.8–89.0
stage * (% of group) I II IIIa IIIb IV	1 (1.9%) 17 (32.7%) 4 (7.7%) 14 (26.9%) 16 (30.8%)	1 (1.4%) 7 (9.7%) 14 (19.4%) 18 (25.0%) 32 (44.4%)	1 (1.0%) 20 (19.8%) 16 (15.8%) 28 (27.7%) 36 (35.6%)
outcome ** cured chronically stable without medication chronically stable with medication progression recurrence follow-up incomplete	24 (46.2%) 16 (30.8%) 10 (19.2%) 1 (1.9%) 0 (0%) 1 (1.9%)	- 8 (11.1%) 57 (79.2%) 7 (9.7%) 0 (0%) -	19 (18.8%) 14 (13.9%) 60 (59.4%) 7 (6.9%) 0 (0%) 1 (1.0%)

* Staging according to WHO-PNM classification of alveolar echinococcosis; in short, stage combines PNM levels, with I - IIIa = localized disease, IIIb - IV = advanced disease [4]. ** Assignment of patients to the respective categories was based on a combination of all diagnostic findings and the interpretation by experienced physicians in the field of alveolar echinococcosis. Prof. Dr. Beate Grüner, as responsible senior physician and head of the section Clinical Infectiology, Steering Group Member of the WHO–IWGE, was in charge.

**Table 2:** Positive ratio of serological markers of interest in N = 59 patients with histopathologically confirmed alveolar echinococcosis.

	Negative	Positive
**recEm18 index**	12 (20.3%)	47 (79.7%)
***Echinococcus* IgG**	4 (6.8%)	55 (93.2%)
**Em2+**	12 (20.3%)	47 (79.7%)
**total IgE**	20 (33.9%)	39 (66.1%)

**Table 3:** Correlations of parameters with imaging, staging, and outcome.

			Tracer Uptake in PET-CTI	Size of Parasitic Lesions	Number of Parasitic Lesions	Initial Staging	Patient Outcome
Spearman-Rho	**recEm18** **index**	K *	0.660 **	0.711 **	0.227 **	0.258 **	0.527 **
		*p*-value	*p* < 0.05	*p* < 0.05	*p* < 0.05	*p* < 0.05	*p* < 0.05
		N	314	368	368	101	123
	***Echinococcus* IgG**	K *	0.670 **	0.697 **	0.287 **	0.321 **	0.502 **
		*p*-value	*p* < 0.05	*p* < 0.05	*p* < 0.05	*p* < 0.05	*p* < 0.05
		N	313	367	367	101	123
	**Em2+**	K *	0.556 **	0.580 **	0.184 **	0.268 **	0.480 **
		*p*-value	*p* < 0.05	*p* < 0.05	*p* < 0.05	*p* < 0.05	*p* < 0.05
		N	312	366	366	101	123
	**total IgE**	K *	0.394 **	0.476 **	0.087	0.186 **	0.272 **
		*p*-value	*p* < 0.05	*p* < 0.05	*p* = 0.05	*p* < 0.05	*p* < 0.05
		N	307	360	360	101	123

* Correlation coefficient, ** correlation is significant *p* < 0.05.

## Data Availability

The data presented in this study are available on request from the corresponding author. The data are not publicly available due to ethical concerns and data privacy reasons.
